# HEALTH INSURANCE AND HEALTH CARE DECISION-MAKING FOR ADULTS IN THE PRE-MEDICARE YEARS: FINDINGS FROM NATIONAL SURVEYS

**DOI:** 10.1093/geroni/igz038.040

**Published:** 2019-11-08

**Authors:** Erica Solway, Renuka Tipirneni, Erica Solway

**Affiliations:** 1 Institute for Healthcare Policy and Innovation, University of Michigan, Ann Arbor, Michigan, United States; 2 University of Michigan Medical School, Ann Arbor, Michigan, United States; 3 Institute for Healthcare Policy and Innovation, University of Michigan, Ann Arbor, Michigan, United States

## Abstract

As more Americans approach retirement age and eligibility for Medicare coverage, many face difficult decisions about their health insurance and health care. This session explores how adults age 50-64 are navigating these choices following implementation of the Affordable Care Act (ACA), presenting data from two nationally representative surveys: The University of Michigan’s National Poll on Healthy Aging (NPHA) and the Health and Retirement Study (HRS). Erica Solway, Associate Director of the NPHA, will begin by presenting background information about the NPHA and an overview of critical health policy issues for adults age 50-64. Jamie Luster, Research Area Specialist at the University of Michigan, will then provide NPHA findings linking concerns about health insurance affordability with delayed/forgone health care. Next, Aaron Scherer, Associate of Internal Medicine at the University of Iowa Carver College of Medicine, will discuss NPHA findings on factors associated with adults’ concern about affordability of health insurance in retirement but before Medicare eligibility begins at age 65. Finally, Renuka Tipirneni, Assistant Professor of Internal Medicine at the University of Michigan, will present findings based on the HRS on changes in health care utilization for adults age 55-64 since implementation of the ACA’s Medicaid expansion. To conclude, Erica Solway will discuss current federal health care policy proposals for adults age 50-64, including the recent introduction of the Medicare at 50 bill, and how the perspectives and experiences of adults in this age group can help inform those policies.

